# Spatial distribution of centromeres and telomeres at interphase varies among *Brachypodium* species

**DOI:** 10.1093/jxb/erv369

**Published:** 2015-07-24

**Authors:** Dominika Idziak, Ewa Robaszkiewicz, Robert Hasterok

**Affiliations:** Department of Plant Anatomy and Cytology, Faculty of Biology and Environmental Protection, University of Silesia in Katowice, 28 Jagiellonska Street, 40-032 Katowice, Poland

**Keywords:** *Brachypodium*, centromeres, interphase, model grass, molecular cytogenetics, Rabl configuration, telomeres.

## Abstract

In this study the 3-D distribution of centromeres and telomeres was analysed in the interphase nuclei of three *Brachypodium* species, i.e. *B. distachyon* (2*n=*10), *B. stacei* (2*n*=20) and *B. hybridum* (2*n*=30), which is presumably a hybrid between the first two species. Using fluorescence *in situ* hybridization (FISH) with centromeric and telomeric DNA probes, it was observed that the majority of *B. distachyon* nuclei in the root tip cells displayed the Rabl configuration while both *B. stacei* and *B. hybridum* mostly lacked the centromere–telomere polarization. In addition, differentiated leaf cells of *B. distachyon* did not display the Rabl pattern. In order to analyse the possible connection between the occurrence of the Rabl pattern and the phase of cell cycle or DNA content, FISH was combined with digital image cytometry. The results revealed that the frequency of nuclei with the Rabl configuration in the root tip nuclei was positively correlated with an increase in DNA content, which resulted from DNA replication. Also, the analysis of the influence of the nuclear shape on the nuclear architecture indicated that an increasing elongation of the nuclei negatively affected the occurrence of the Rabl pattern. Some possible explanations of these phenomena are discussed.

## Highlight

A 3-D analysis of *Brachypodium* interphase nuclei reveals varying patterns of centromere and telomere distribution among closely related species and indicates the factors that may influence the nucleus architecture.

## Introduction

The analysis of spatial architecture of the interphase nucleus has been of interest to biologists for nearly 130 years. Since the pioneering works of Rabl (1885) and Boveri (1909), the relationship between nuclear structure and function has become the focal point of numerous studies. Many of these have linked the regulation of the spatio-temporal genome function with the global arrangement of interphase chromatin into distinct chromosome territories (for recent reviews see Schneider and Grosschedl, 2007; Cremer and Cremer, 2010; Schubert and Shaw, 2011). The territorial organization of chromosomes in interphase nuclei was already being postulated as early as the late 19th century (Rabl, 1885). It took nearly 100 years before convincing experimental support of this hypothesis was provided by Cremer *et al.* (1982), who demonstrated that UV irradiation of a small part of the interphase nucleus damaged discrete chromosomal regions. The development of *in situ* hybridization-based chromosome painting delivered further evidence for the existence of chromosome territories (CTs) in the nuclei of human (Cremer *et al.*, 1988; Pinkel *et al.*, 1988; Croft *et al.*, 1999; Cremer and Cremer, 2001), some animals (Stadler *et al.*, 2004; Neusser *et al.*, 2007; Koehler *et al.*, 2009), and plants (Lysak *et al.*, 2001; Pecinka *et al.*, 2004; Idziak *et al.*, 2011). Many analyses of relative positions of interphase chromosomes indicated non-random radial arrangement of the CTs. The nuclear localization of particular CTs was shown to be influenced by such factors as gene density and chromosome size in the nuclei of human (Boyle *et al.*, 2001; Cremer and Cremer, 2001), various primates and Old-World monkeys (Tanabe *et al.*, 2002; Neusser *et al.*, 2007), as well as chicken and mouse (Habermann *et al.*, 2001; Mayer *et al.*, 2005). By contrast, the distribution of CTs in the model dicotyledonous plant *Arabidopsis thaliana* seems to be random with the exception of the chromosomes that bear the nucleolar organizing region, which display a tendency to associate (Pecinka *et al.*, 2004).

An alternative to the radial positioning of chromosome domains is the arrangement in which all of the centromeres are grouped close to the nuclear periphery at one side of the nucleus while the telomeres are gathered in the opposite nuclear hemisphere (Dong and Jiang, 1998; Cowan *et al.*, 2001). Such a polarized organization of interphase chromatin, also known as the Rabl configuration, commonly occurs in plants but is rarely seen in mammals (Parada and Misteli, 2002). The notable exceptions are fibroblasts in the tree shrew (Haaf and Ward, 1995) and Indian muntjac lymphocytes (Sperling and Ludtke, 1981). Both fission (Funabiki *et al.*, 1993) and budding (Jin *et al.*, 1998) yeast also display a Rabl-like spatial organization of chromatin with the centromeres forming a tight cluster near the nuclear periphery.

The Rabl configuration is a direct consequence of the arrangement of the sister chromatids that segregate at anaphase and can be considered to be the ‘default’ organization of the interphase chromatin. However, this division-related configuration is retained at the following interphase only in some species, while in others a post-mitotic chromosome decondensation leads to the loss of their polarized orientation (Cowan *et al.*, 2001; Santos and Shaw, 2004). A comparative analysis of centromere and telomere distribution in a range of cereals showed that species that have large (>4800Mb) genomes, such as wheat, barley, rye, and oats, display the Rabl configuration in their nuclei, while species like sorghum and rice, all with genomes <1000Mb, do not (Dong and Jiang, 1998). These data led to the conclusion that the presence of the Rabl configuration depends on nuclear genome size. However, this hypothesis can be challenged, since some small genome organisms, e.g., yeast and *Drosophila*, clearly show the Rabl pattern (Hochstrasser *et al.*, 1986). Concurrently, this configuration is largely absent in mammalian cells that have relatively large genomes (Cowan *et al.*, 2001).

Another explanation associates the presence of the Rabl configuration with the length of chromosomes and the content and distribution of heterochromatin. In species like wheat or barley, which have a high content of heterochromatin that is distributed uniformly along the entire length of chromosomes, the process of chromatin decondensation probably only affects specific regions that contain euchromatin. Therefore large, mostly heterochromatic chromosomes are subjected only to relatively minor conformational changes. However, if the heterochromatin concentrates around centromeres, as in *A. thaliana* and many other species with small chromosomes, chromatin decondensation at the onset of interphase can result in a significantly different conformation of the predominantly euchromatic chromosome arms (Santos and Shaw, 2004).

In the present study, the distribution of the centromeres and telomeres has been characterized in *B. distachyon*, *B. stacei*, and *B. hybridum*, which is presumably a hybrid of the first two species. Because all the aforementioned species are characterized by a small genome size below 620Mb/1C, it was assumed that, based on the genome size rule proposed by Dong and Jiang (1998), all of them would lack the Rabl arrangement of chromosomes. Surprisingly, it was observed that *B. distachyon* displays a Rabl configuration in the nuclei in the root tip while both *B. stacei* and *B. hybridum* lack the centromere–telomere polarization in their root-tip cells. In addition, differentiated leaf cells of *B. distachyon* do not display the Rabl pattern. Some possible explanations of these phenomena are suggested.

## Materials and methods

### Plant material

Three different *Brachypodium* species were analysed in this study. Seeds were obtained from the collections held by Aberystwyth University (UK) and USDA-NPGS. Detailed information on the origin and chromosome numbers of the material is provided in Table 1.

**Table 1.** Origins, chromosome numbers, and accession details of the Brachypodium material used in this study

Species	Accession no.	Origin	2*n*	Ploidy level
*B. distachyon*	Bd21	Iraq	10	Diploid
*B. stacei*	ABR114	Spain	20	Diploid
*B. hybridum*	ABR113	Portugal	30	Allotetraploid

### Preparation of nuclei

Seeds were grown in Petri dishes on filter paper that had been moistened with tap water for 2–3 d at room temperature in the dark. Whole seedlings with roots 1.5–2cm long were collected and fixed in 4% formaldehyde in PBS (pH 7.3) for 30min on ice. Nuclei isolation was adopted from Dolezel *et al.* (1989). For the preparation of the slides, roots were cut off from seedlings, washed in PBS (3×10min, 0 °C), then washed in TRIS buffer (10mM TRIS–HCl, pH 7.5, 10mM Na_2_-EDTA, 100mM NaCl) for 20min and chopped with a razor blade in LB01 buffer (15mM TRIS–HCl, pH 7.5, 2mM Na_2_-EDTA, 0.5mM spermine·4HCl, 80mM KCl, 20mM NaCl, 0.1% Triton X-100) in a Petri dish on ice. The suspension of nuclei was filtered through a mesh filter with a pore size of 30 μm and dropped on to microscopic slides that had been cooled to about 0 °C. In the case of leaf tissue, the suspension of nuclei was additionally centrifuged (700g, 4 °C, 2×3min) in 0.1% Triton X-100 in 1×PBS to remove the chloroplasts. The slides were air-dried and stored at –20 °C until use.

### Probe labelling and fluorescence *in situ* hybridization

The following DNA sequences were used as probes.

(i) HT100.3: *Arabidopsis*-like telomeric repeats (TTTAGGG)_n_ labelled using PCR with tetramethylrhodamine-5-dUTP (Roche).
(ii) A centromeric BAC (Bacterial Artificial Chromosome) clone CB33J12 that had originated from the *B. distachyon* genomic DNA sequencing library (Febrer *et al.*, 2010; IBI, 2010), which was isolated using the standard alkaline extraction as described by Farrar and Donnison (2007) and subsequently labelled by nick-translation using digoxigenin-11-dUTP (Roche).

The details of probe labelling and the following fluorescence *in situ* hybridization (FISH) procedure were as described by Jenkins and Hasterok (2007) with minor modifications. Centromeric and telomeric probes were mixed together, precipitated, and dissolved in a hybridization mixture containing 50% deionized formamide and 10% dextran sulphate in 2× saline sodium citrate (SSC). After denaturation (10min, 75 °C), the hybridization mixture was applied to slides with isolated nuclei and denatured again at 75 °C for 4.5min. Hybridization was performed in a humid chamber at 37 °C for about 40h. After hybridization, the slides were washed in 10% formamide in 2× SSC (2×4min, 42 °C), which is equivalent to a 79% stringency. Immunodetection of the probes that had been labelled with digoxigenin-11-dUTP was performed according to the standard protocols using fluorescence isothiocyanate (FITC)-conjugated anti-digoxigenin antibodies (Roche). The slides were mounted in Vectashield (Vector Laboratories) containing 2.5 μg ml^–1^ DAPI (Serva).

### Image acquisition, processing, and analysis

All images of the interphase nuclei were acquired using an Olympus FV1000 confocal system (Olympus, Poland) or a Zeiss Axio Imager.Z.2 wide-field fluorescence microscope equipped with an AxioCam Mrm monochromatic camera and Apotome.2 system. Image processing operations (including z-stacks rendering in 3-D) and the construction of 3-D models of nuclei using ‘Spot detection’ and ‘Contour surface’ wizards were performed with Imaris software (Bitplane). All nuclei were analysed individually and ‘manually’ after reconstructing the 3-D image from the optical sections. Our methodological approach ensured that the detection of Rabl configuration was not affected by the shape of the nucleus. The distribution of centromeres and telomeres was assessed while rotating the nucleus in different directions to check if the polarization is present along any of the axes.

Quantitative acquisition and analysis were performed using a high-content screening system (Scan^R, Olympus) based on a wide-field microscope (Olympus IX81) that was equipped with a CCD camera (Hamamatsu ORCA-ER) and an MT20 illumination system (Xenon-mercury lamp, 150W). Threshold values were used to perform the automatic segmentation of nuclei. The segmentation of the nuclei into the G1 phase, G2 phase or endoreplication phase (E) was based on total DAPI fluorescence intensities (the sum of the pixel intensity value that was specific for the object). Within each class, the nuclei were further divided into subclasses according to their shape (spherical, elongated or rod-shaped) and the percentage of nuclei with and without Rabl configuration was established.

## Results

### The Rabl arrangement occurs in the majority of *B. distachyon* root tip nuclei

The analysis of the root tip nuclei that were isolated from *B. distachyon* seedlings revealed considerable diversity of nuclear shapes and sizes. The morphology of the nuclei varied from spherical through elongated to a very long, rod-like shape, with the first two shape types being the most common. The nuclear volume was estimated for 100 nuclei. For 75% of the nuclei that were analysed, it ranged from 70 μm^3^ to 250 μm^3^. The other 25% comprised predominantly larger nuclei with a volume reaching over 1 100 μm^3^. This volume variation could be attributed to the differences in the DNA content between the nuclei in G1, S, and G2 phases of the mitotic and endoreduplicated cell cycle, as had previously been proved for several angiosperm species by Jovtchev *et al.* (2006).

**Fig. 1.**
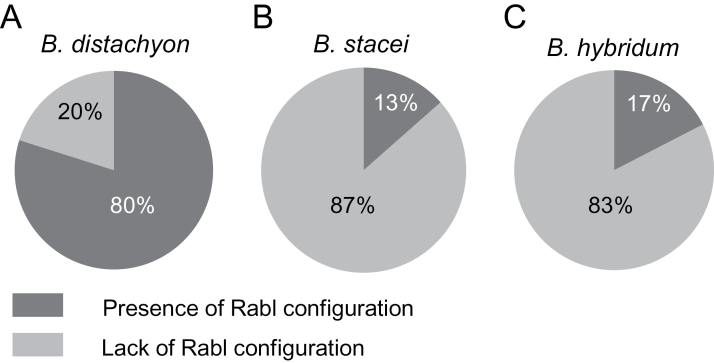
The percentage of the root tip nuclei that displayed the presence or lack of the Rabl-like arrangement of the centromeres and telomeres in (A) *B. distachyon*, (B) *B. stacei*, and (C) *B. hybridum.*

**Fig. 2.**
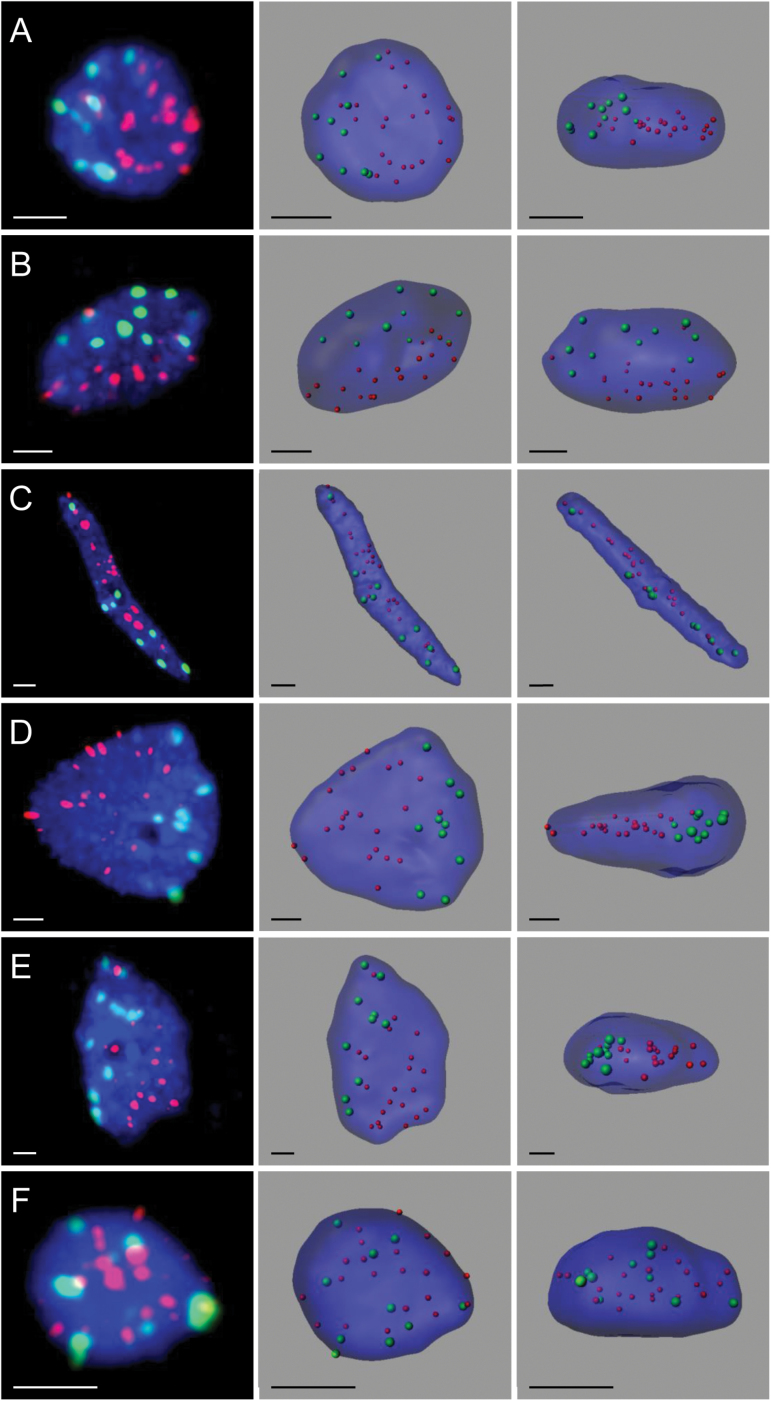
The organization of the centromeres and telomeres in the *B. distachyon* root tip (A–E) and leaf (F) nuclei. All nuclei presented except (F) display the Rabl configuration. The left column shows the FISH results using centromeric (green signals) and telomeric (red signals) sequences as probes. Chromatin is stained with DAPI (blue fluorescence). The middle and right columns present the nuclei structures modelled using FISH results and observed in two different planes. (A) Small spherical nuclei, (B) small elongated nuclei, (C) rod-shaped nuclei, (D) spherical endoreduplicated nuclei, and (E) elongated endoreduplicated nuclei. Bars: 2 μm.

In order to investigate the distribution of centromeres and telomeres in the *B. distachyon* root tip nuclei, the centromere-specific BAC clone CB33J12 and the telomeric probe HT100.3 were used. Two patterns of the spatial arrangement of centromeres and telomeres were observed (Figs 1A, 2 A–E; see Supplementary Fig. S1 and Supplementary Videos S1–S10 at *JXB* online). No Rabl-like arrangement was observed in about 20% of the nuclei as the centromeres and telomeres were scattered more or less uniformly across the entire nuclear space (see Supplementary Fig. S1 and Supplementary Videos S6–S10 at *JXB* online). The majority of nuclei displayed the Rabl configuration with clear separation of the telomeric and centromeric sequences in two different areas of the nucleus (Fig. 2A–E; see Supplementary Videos S1–S5 at *JXB* online). The telomeres and centromeres did not form tight clusters at the opposite poles but were dispersed within their relevant zones. The area that was occupied by the telomeres was usually slightly larger than the area that contained the centromeric sequences. The intermediate stage between the polarized and dispersed distribution of centromeres and telomeres was not observed. The two areas comprising each type of sequences in Rabl nuclei were clearly discriminated. Despite a slight flattening of the isolated nuclei during the FISH procedure, they still provided a good substrate to observe the FISH signals in 3-D at different angles. Nevertheless, in order to minimize the risk of misclassification, the most flattened nuclei were excluded from the analysis. The results showed that the distribution of centromeres and telomeres could be polarized in various directions in different nuclei (Fig. 2A; see Supplementary Fig. S2 and Supplementary Videos S1–S5 and S11 at *JXB* online). Frequently, the polarization of the nuclei that displayed the Rabl configuration was disrupted by the presence of one to two telomeric signals within the area that was occupied by centromeres (Fig. 2A–E; see Supplementary Videos S1–S5 at *JXB* online). These signals usually localized close to one or two of the centromeres, which might indicate that they belonged to the acrocentric chromosome 5.

### The distribution of centromeres and telomeres in *B. distachyon* root tip nuclei is affected by the nuclear shape

**Fig. 3.**
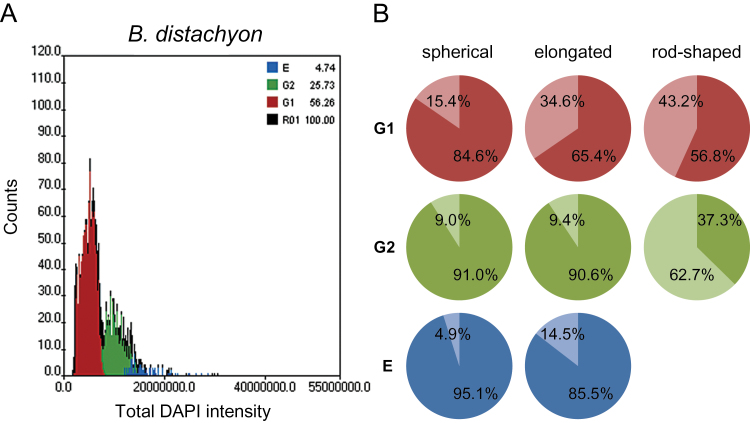
Analysis of the influence of nuclear shape and DNA content on the nucleus architecture. (A) Histograms showing the quantitative analysis of the root tip nuclei of *B. distachyon*. The nuclei were gated into the G1, G2, and endoreduplication [E] phases based on their total DAPI intensity. (B) The percentage of *B. distachyon* root tip nuclei that displayed the presence (darker shade of colour) or lack (lighter shade of colour) of the Rabl configuration, based on their nuclear shape and DNA content.

In order to determine whether the presence of the Rabl configuration in the *B. distachyon* root tip nuclei corresponds with the cell cycle phase or DNA content, digital image cytometry using an Olympus Scan^R screening system was used to analyse a representative sample of over 1 000 nuclei and gate them into the G1, G2, and endoreduplication (E) phases based on their total DAPI fluorescence intensities (Fig. 3A). The three populations of the nuclei (G1, G2, and E) were then further divided into three categories according to their nuclear shape (Fig. 3B). The width (*d*) and length (*l*) of nuclei projected along the *z* axis were measured and the *l*/*d* proportion was calculated for each nucleus. The nuclei were then classified as spherical, elongated or rod-shaped based on the *l*/*d* factor value, which ranged from 1.0 to 1.4, 1.5 to 3.5, and over 3.5, respectively. The results showed that spherical and elongated nuclei usually displayed the Rabl arrangement independent of the cell cycle phase (Figs 2A, B, D, E, 3B). The percentage of spherical and elongated nuclei with the Rabl configuration increased in G2 and after endoreduplication compared with the G1 phase (Fig. 3B). The association between the increased occurrence of the Rabl pattern and the increase in DNA content was estimated using the Pearson’s Chi-squared test of independence and was found to be highly significant (χ^2^=30.41; *P* <0.001).

The analysis of the influence of nuclear shape on nuclear architecture indicated that the less spherical the nuclei, the lower the frequency of the polarized distribution of telomeres and centromeres. The Rabl configuration was observed in 84.6% and 65.4% of G1 spherical and elongated nuclei, respectively, and in only 56.8% of G1 rod-shaped nuclei. This tendency was also noted in the G2 phase in which the nuclei that displayed the Rabl arrangement constituted 37.3% of the rod-shaped nuclei population compared with 91.0% and 90.6% values for spherical and elongated nuclei, respectively. Interestingly, no rod-shaped nuclei were observed within the population of endoreduplicated nuclei. The nuclei with the polarization of centromeres and telomeres constituted over 95% of spherical endoreduplicated nuclei. The analogical value that was estimated for the elongated nuclei after endoreduplication was 85.5% (Fig. 3B). All endoreduplicated nuclei (Fig. 2D, E) were significantly larger than the G1 and G2 nuclei (Fig. 2A, B) and could even be recognized in the preparations without the digital image cytometry analysis data. The hypothesis that the nuclear shape might be a factor that influences nuclear architecture was confirmed by the Chi-squared statistics with a very high confidence level (χ^2^=30.41, *P* <0.001). The statistical significance of that influence was also proved when each category of nuclei was tested separately (χ^2^=25.91; χ^2^=116.55, and χ^2^=77.65 for the G1, G2, and endoreduplicated nuclei, respectively; *P* <0.001).

### *B. distachyon* leaf nuclei do not display the Rabl configuration

The analysis of telomere and centromere distribution using FISH was also performed on isolated leaf nuclei. Only one type of nucleus was observed. The leaf nuclei were spherical, brightly stained with DAPI, and significantly smaller than the spherical nuclei of the root tip. Surprisingly, no Rabl configuration was detected in any of the nuclei that were analysed and the centromeres and telomeres were dispersed within the nuclear space in all of the nuclei (Fig. 2F; see Supplementary Video S12 at *JXB* online). Frequently, several telomeric signals were observed in the peripheral area of the nucleus. In contrast to the root tip nuclei in which ten centromeres were repeatedly observed, the leaf nuclei displayed a varying number of centromeric signals from five to ten (see Supplementary Fig. S3 at *JXB* online). The most frequent numbers of signals that were detected was eight (32.8%), seven (26.6%), and six (26.6%). Ten signals were observed in only 4.7% of the leaf nuclei. In the cases in which fewer than ten centromeric signals were observed, some of them were significantly larger than the others, possibly due to the centromere association (data not shown).

### *B. stacei* and *B. hybridum* root tip nuclei generally lack the Rabl configuration

**Fig. 4.**
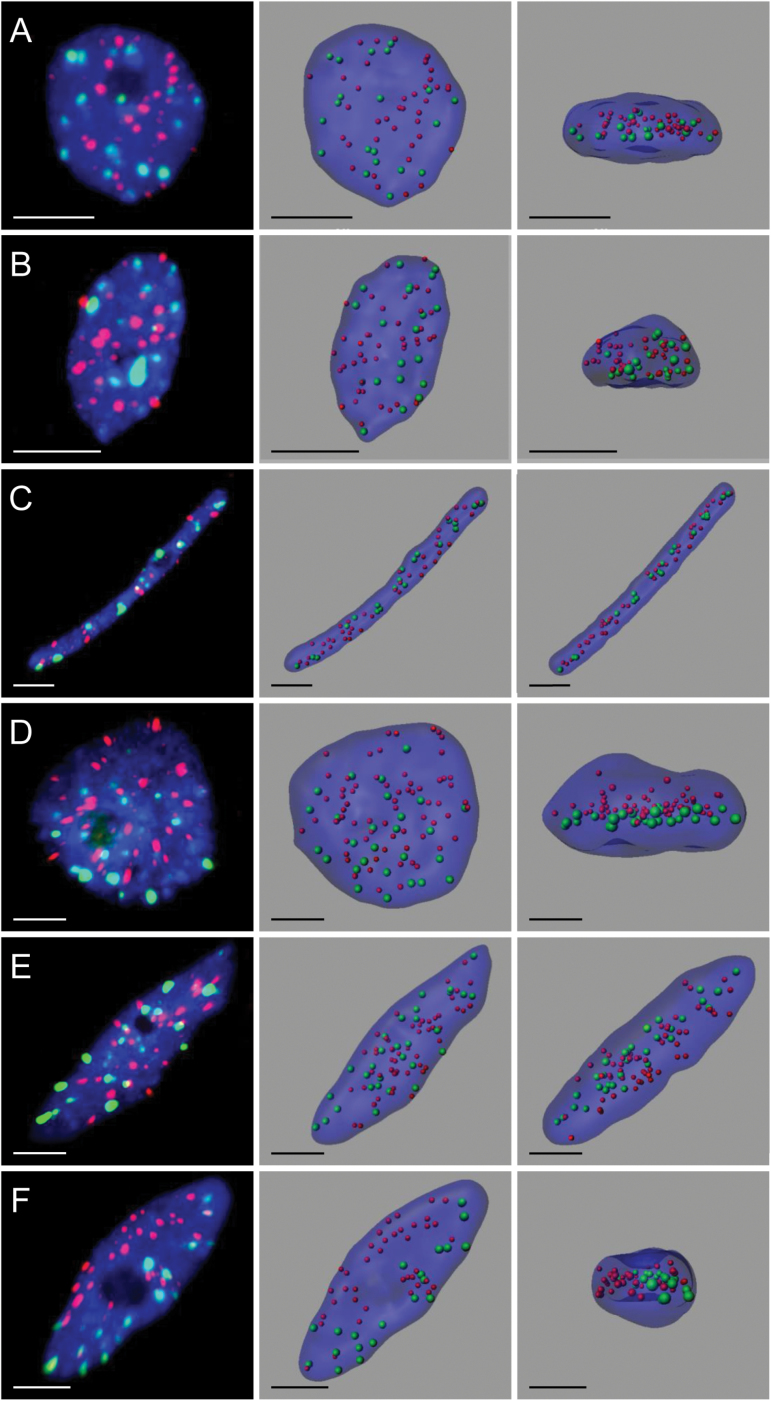
The organization of the centromeres and telomeres in *B. stacei* (A–C, F) and *B. hybridum* (D, E) root tip nuclei. All the nuclei presented, except (D) and (F), display a lack of the Rabl configuration. The left column shows the FISH results using centromeric (green signals) and telomeric (red signals) sequences as probes. Chromatin is stained with DAPI (blue fluorescence). The middle and right columns present the nuclei structures modelled using FISH results and observed in two different planes. (A) Small spherical nuclei, (B) small elongated nuclei, (C) rod-shaped nuclei, (D) spherical endoreduplicated nuclei, (E) and (F) elongated endoreduplicated nuclei. Bars: 5 μm.

In addition to *B. distachyon*, two other *Brachypodium* species—*B. stacei* (2*n*=20) and *B. hybridum* (2*n*=30), were included in the analyses. The latter species is an allopolyploid that originated from the hybridization of *B. distachyon* and *B. stacei*. The root tip nuclei that were observed in *B. stacei* and *B. hybridum* were slightly larger than *B. distachyon* nuclei. The size difference was particularly noticeable in the case of the polyploid. Similar to *B. distachyon*, three types of nuclear shape were discriminated in both of the species that were investigated—spherical, elongated, and rod-like (Fig. 4).

Mapping the centromeric and telomeric sequences to the root tip nuclei of *B. stacei* and *B. hybridum* revealed that, in contrast to *B. distachyon*, these two species generally lack the Rabl configuration (Figs 1B, C, 4 A–C, E; see Supplementary Videos S13–S16 at *JXB* online). The percentage of nuclei with a polarized distribution of centromeres and telomeres equalled 13% and 17% for *B. stacei* and *B. hybridum*, respectively. However, even in those nuclei, the centromeric and telomeric sequences often intermingled at the borders of their relevant zones, which led to the disruption of the Rabl arrangement (Fig. 4D, F; see Supplementary Videos S17 and S18 at *JXB* online). The Chi-squared test of independence showed that the differing level of nuclei that display the Rabl pattern in *B. distachyon*, *B. stacei*, and *B. hybridum* is significant (χ^2^=211.76; *P* <0.001).

**Fig. 5.**
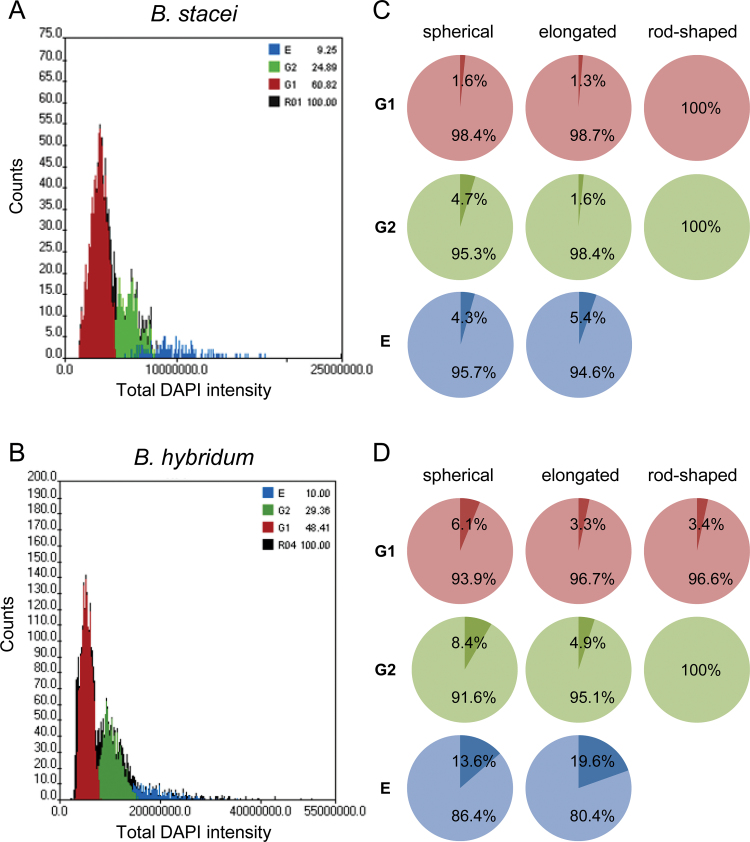
Analysis of the influence of nuclear shape and DNA content on the nucleus architecture. (A, B) Histogram showing the quantitative analysis of root tip nuclei of *B. stacei* (A) and *B. hybridum* (B). The nuclei were gated into the G1, G2, and endoreduplication [E] phases based on their total DAPI intensity. (C, D) The percentage of *B. stacei* (C) and *B. hybridum* (D) root tip nuclei that displayed the presence (darker shade of colour) or lack (lighter shade of colour) of Rabl configuration, based on nuclear shape and DNA content.

The analysis of nuclei populations using digital image cytometry suggested that the same factors that influenced the centromere and telomere distribution in *B. distachyon* also affected the occurrence of the Rabl pattern in *B. stacei* and *B. hybridum*. The frequency of nuclei with the Rabl configuration increased with an increase in DNA content in spherical and elongated nuclei. In G1 and G2, it decreased with the increasing elongation of the nuclei (Fig. 5). Using the Chi-squared test with the cutoff of *P* <0.1, statistical analysis seems to support the influence of the DNA content on the occurrence of the Rabl configuration. However, the hypothesis that the occurrence of the Rabl pattern is negatively affected by the increased elongation of the nuclei could not be proved by the Chi-squared test using the same level of confidence. The reason is the lack of nuclei with the Rabl configuration within the rod-shaped nuclei populations in the G1 and G2 phases in *B. stacei* and the G2 phase in *B. hybridum*.

A common feature of both species, which they also shared with *B. distachyon*, was the lack of rod-shaped nuclei among the endoreduplicated nuclei. The highest number of nuclei that displayed the Rabl configuration was found in endoreduplicated elongated nuclei—5.4% in *B. stacei* (Fig. 5A, B) and 19.6% in *B. hybridum* (Fig. 5C, D). The comparison between the two species showed that, in each nuclei category that was based on the shape and the cell cycle phase, the percentage of nuclei with the Rabl arrangement was consistently higher in *B. hybridum* than in *B. stacei* (Fig. 5B, D).

## Discussion

The increased interest in the analysis of nuclear architecture arises from the widely accepted consensus that a three-dimensional distribution of interphase chromatin has major implications in such fundamental processes as DNA replication, transcription, and repair (Heard and Bickmore, 2007; Misteli, 2007; Misteli and Soutoglou, 2009; Mekhail and Moazed, 2010).

However, despite numerous studies on the connection between nucleus structure and function, the mechanisms that are responsible for chromatin reorganization as a response to the current needs of a cell are not yet well understood. In addition, all the factors that decide how a certain pattern of nuclear organization is established and maintained are not known. Genome size is at the top of the list of factors that presumably influence nucleus structure in plants. Dong and Jiang (1998) postulated that a high nuclear DNA content promotes the presence of the Rabl configuration. This proved to be true for species with a genome size exceeding 4 800 Mbp such as, for example, wheat, barley (Dong and Jiang, 1998), or *Vicia faba* (Rawlins *et al.*, 1991). The fact that most of small genome species that have been analysed to date exhibit a non-Rabl pattern with telomeres and centromeres rather uniformly dispersed within the entire nuclear space appears to corroborate this hypothesis. The nuclei of *A. thaliana*, which display a non-Rabl configuration with telomeres that are clustered around a nucleolus and centromeres that are widely dispersed and located peripherally are a notable deviation from this arrangement (Armstrong *et al.*, 2001; Fransz and de Jong, 2002). No Rabl configuration has been observed in maize, which has an intermediate genome size (~3 000Mb), but the positioning of centromeres and telomeres was not entirely random either (Dong and Jiang, 1998).

The three species of *Brachypodium* that were used in our study have relatively small genomes. The smallest one belongs to *B. stacei* (0.282 pg≈276Mb/1C DNA) while *B. distachyon* has a genome size of 0.316 pg≈309Mb [pg to Mb conversion made according to Dolezel *et al.* (2003)]. The DNA content of an allotetraploid *B. hybridum* is 0.633 pg≈619Mb, which roughly approximates the sum of the genome sizes of its putative parents (Wolny and Hasterok, 2009; Catalan *et al.*, 2012). Surprisingly, despite the similarity in genome size, the dominant patterns of centromere and telomere distribution differ significantly among the three species. While *B. stacei* and *B. hybridum* follow the genome size rule that was established by Dong and Jiang (1998), *B. distachyon* root tip nuclei mostly display the Rabl configuration, thus indicating that, in the case of these species, other factors that determine the nucleus structure come into play. The most significant differences between the genomes of *B. distachyon*, *B. stacei*, and *B. hybridum* at the cytological level are the number and size of the chromosomes. The 20 chromosomes of *B. stacei* are considerably smaller than the 10 chromosomes of *B. distachyon*. The karyotype of the hybrid comprises 10 large and 20 small chromosomes that are derived from the parental species (Hasterok *et al.*, 2004). It has been argued that small chromosomes can change their conformation more easily than large ones which are more likely to preserve their anaphase arrangement after entering the following interphase (Dong and Jiang, 1998). It is possible then that the relatively large *B. distachyon* chromosomes are more inclined to keep occupying ordered elongated territories while the small chromosomes of *B. stacei* disperse more freely inside the nucleus. In such a case, one could presume that, in *B. hybridum*, the centromere–telomere polarization imposed by larger chromosomes is disrupted by small chromosomes that are scattered among them, thus resulting in a predominantly non-Rabl pattern.

Besides the variation in the nuclear spatial arrangement between such closely related species with a similar DNA content, another unexpected outcome was the difference in the nucleus structure that was observed between the leaf and root tip cells of *B. distachyon*. Previous studies on seven crop species had indicated that the distribution pattern of centromeres and telomeres was uniform and conserved in the leaf, root tip, and pre-meiotic cells (Dong and Jiang, 1998). Rice pre-meiotic and root xylem vessel cells that displayed the Rabl pattern, unlike the rest of the rice tissues, were the one deviation from this uniformity (Prieto *et al.*, 2004). The tissue-specificity of the chromatin arrangement is more frequently observed in animals. In *Drosophila* embryos, 100% of nuclei are characterized by telomere and centromere polarization (Marshall *et al.*, 1996) while, in *Drosophila* salivary glands, only 80% of nuclei were shown to have Rabl configuration (Hochstrasser *et al.*, 1986). In mice, different cerebellar cell types display distinct distribution patterns of centromeres, which can be associated with the nuclear envelope or clustered around the nucleolus (Manuelidis, 1984). Differential, tissue-specific positioning of chromosomes was also found in mouse lymphocytes as well as in liver and lung cell nuclei (Parada *et al.*, 2004). This clearly indicates that the spatial organization of chromatin in the nucleus may play an important role in tissue differentiation. It is possible then that the non-Rabl configuration by *B. distachyon* leaf nuclei is a part of the mesophyll cell differentiation programme. Alternatively, the presence of the Rabl configuration in the majority of the root tip nuclei might be associated with the presence of rapidly dividing cells in the meristematic and elongation zones of the growing root. There has been speculation that the Rabl pattern helps chromosomes to achieve their condensed metaphase state (Cowan *et al.*, 2001). It was demonstrated that chromosomes do not undergo much internal reorganization at the G2–M transition in muntjac cells. As a result, the structure of the prophase chromosome territory relates directly to that of an interphase chromosome (Manders *et al.*, 1999). The significance of the Rabl configuration for proper chromosome segregation in mitosis is further supported by recent studies in fission yeast in which analyses of a novel protein, Csi1, provided evidence that centromere clustering directly contributes to the efficient capture of kinetochores by microtubules (Hou *et al.*, 2012, 2013). Thus, the polarization of centromeres and telomeres in the root tip nuclei of *B. distachyon* might facilitate mitotic cell divisions. It would not then be needed by the fully differentiated leaf cells that enter the G0 phase and do not divide anymore. Over time, the centromeres and telomeres in the leaf nuclei would become uniformly dispersed within the nuclear space. Some feedback for this hypothesis can be obtained from the research on budding yeast in which centromere clustering was found to occur in 90% of the cells in growing cultures but was significantly reduced in the cells that had been kept under stationary conditions (Jin *et al.*, 1998).

Although a dominant pattern of the nucleus structure was discerned for the root tip nuclei of all three of the species that were analysed, the nuclei populations displayed a certain level of heterogeneity in terms of centromere and telomere distribution. The detailed data about the percentage of nuclei with Rabl or non-Rabl configuration in a given tissue are scarce. In most publications concerning plant species it is usually stated that almost all or the majority of nuclei display a certain pattern of nuclear organization (Rawlins *et al.*, 1991; Dong and Jiang, 1998). Thus, a mixed proportion of Rabl and non-Rabl nuclei is to be expected, but the dominant pattern of centromere and telomere distribution is easy to discern. The diversity within a population of nuclei that had been derived from the same plant organ can be attributed to the varying cell differentiation programmes. The specialization of the cell manifests itself *inter alia* through changes in the cell shape and, by extension, the changes in the shape of the nucleus. The three types of nuclei that were observed in the root tips of all of the *Brachypodium* species are also found in *Arabidopsis* (Chytilova *et al.*, 1999; Pecinka *et al.*, 2004). The spherical shape of the nuclei is characteristic for meristematic or undifferentiated cells while elongated nuclei are found in the root epidermis and rod-shaped nuclei are specific to vascular tissues (Chytilova *et al.*, 1999). The nucleus shape was found to be an influential factor in determining the position of centromeres and telomeres. The increasing elongation of the nuclei negatively affected the frequency of the occurrence of the Rabl pattern. It implies that the change from the spherical shape to the highly elongated one imposes some constraints on the distribution of chromatin within the nucleus, which results in a loss of the Rabl configuration.

The dependence of spatial chromatin arrangement on the cell cycle stage has been shown many times in past and recent works (Ferguson and Ward, 1992; Csink and Henikoff, 1998; Solovei *et al.*, 2004; Grand *et al.*, 2014). In *Drosophila*, the Rabl configuration was an attribute of cells in the G1 stage and it dispersed quickly as the G1 stage progressed to the S and G2 stages (Csink and Henikoff, 1998). It was assumed that this could also have been one of the reasons for the diversity of the centromere and telomere distribution between the root tip nuclei of *Brachypodium*. However, no such correspondence was revealed by digital image cytometry. Instead, a slight increase in the number of cells that displayed the Rabl pattern was found in the G2 cells of all the species analysed. It is possible that this phenomenon is related to the increase in DNA content, since the percentage of nuclei with the Rabl configuration was even higher among the endoreduplicated cells. The association of the centromeres that is induced by polyploidy has been demonstrated for several Triticeae species (Martinez-Perez *et al.*, 2000). As mentioned previously, the rice xylem vessel precursor cells were found to be the only somatic cells to adopt the Rabl configuration (Santos and Shaw, 2004). Since xylem vessel cells are known to undergo endoreduplication (Martinez-Perez *et al.*, 2001), the presence of the Rabl pattern was attributed to the interactions between the centromeres of endoreduplicated chromosomes (Santos and Shaw, 2004). It is possible that a similar mechanism contributed to the increased number of endoreduplicated nuclei that had telomere and centromere polarization in the root tips of *Brachypodium* species.

Despite all of the data that has been gathered to date, there is no satisfying answer to the question as to what the exact role of the Rabl configuration is, nor what the factors that determine its presence or absence are. One of the hypotheses states that a non-random orientation of chromosomes might contribute to the formation of ‘transcription factories’ in which active genes are recruited for their expression (Iborra *et al.*, 1996; Schoenfelder *et al.*, 2010; Tanizawa *et al.*, 2010). In cereals such as wheat and barley, but also in many other organisms, the gene density increases towards the chromosome termini, while their proximal regions remain relatively gene poor. Therefore, it can be assumed that the Rabl arrangement would facilitate the formation of ‘transcription factories’ by bringing the distal, gene-rich parts of chromosomes closer to each other (Schubert and Shaw, 2011). However, the BrdU labelling of nascent transcripts in wheat nuclei showed that there is no preferential positioning of the active transcription sites that would reflect the gene-density gradient (Abranches *et al.*, 1998). Judging from these results, the Rabl pattern does not appear to contribute to the spatial organization of gene transcription.

The importance of the Rabl configuration in facilitating mitotic cell division was discussed above. In addition, it may also play a role in the nuclei at the onset of meiosis. One hypothesis associates the possible function of the Rabl orientation with the alignment of homologous chromosomes in meiosis through facilitating the clustering of telomeres into a bouquet structure (Parada and Misteli, 2002). However, the bouquet formation in maize does not require a pre-existing Rabl configuration (Bass *et al.*, 1997). Recent study on *B. distachyon* meiosis showed that the centromeres are dispersed across the whole nucleus in the early pre-meiotic pollen mother cells but form a tight cluster at the onset of meiosis (Wen *et al.*, 2012). Moreover, some organisms display only one type of structure—a Rabl configuration or a bouquet—without displaying the other (Cowan *et al.*, 2001). In order to elucidate the significance of the Rabl orientation fully, comprehensive studies would be required that would address simultaneously the criteria that determine the presence of the Rabl configuration and the mechanisms that are involved in its maintenance.

## Supplementary data

Supplementary data can be found at *JXB* online.

Supplementary Fig. S1. The *B. distachyon* root nuclei that displayed the lack of the Rabl configuration.

Supplementary Fig. S2. The Rabl organization of the centromeres and telomeres in *B. distachyon* in the small spherical nuclei of the root tip observed in two different planes.

Supplementary Fig. S3. The percentage of *B. distachyon* leaf nuclei that displayed a varying number of centromere signals.

Supplementary Video S1. The Rabl organization of the centromeres and telomeres in *B. distachyon* in the small spherical nucleus in the root tip that is presented in Fig. 2A.

Supplementary Video S2. The Rabl organization of the centromeres and telomeres in *B. distachyon* in the small elongated nucleus of the root tip that is presented in Fig. 2B.

Supplementary Video S3. The Rabl organization of the centromeres and telomeres in *B. distachyon* in the rod-shaped nucleus of the root tip that is presented in Fig. 2C.

Supplementary Video S4. The Rabl organization of the centromeres and telomeres in *B. distachyon* in the spherical endoreduplicated nucleus of the root tip that is presented in Fig. 2D.

Supplementary Video S5. The Rabl organization of the centromeres and telomeres in *B. distachyon* in the elongated endoreduplicated nucleus of the root tip that is presented in Fig. 2E.

Supplementary Video S6. The non-Rabl organization of the centromeres and telomeres in *B. distachyon* in the small spherical nucleus of the root tip that is presented in Supplementary Fig. 1A.

Supplementary Video S7. The non-Rabl organization of the centromeres and telomeres in *B. distachyon* in the small elongated nucleus of the root tip that is presented in Supplementary Fig. 1B.

Supplementary Video S8. The non-Rabl organization of the centromeres and telomeres in *B. distachyon* in the rod-shaped nucleus of the root tip that is presented in Supplementary Fig. 1C.

Supplementary Video S9. The Rabl organization of the centromeres and telomeres in *B. distachyon* in the spherical endoreduplicated nucleus of the root tip that is presented in Supplementary Fig. 1D.

Supplementary Video S10. The non-Rabl organization of the centromeres and telomeres in *B. distachyon* in the elongated endoreduplicated nucleus of the root tip that is presented in Supplementary Fig. 1E.

Supplementary Video S11. The Rabl organization of the centromeres and telomeres in *B. distachyon* in the small spherical nucleus of the root tip that is presented in Supplementary Fig. 2.

Supplementary Video S12. The non-Rabl organization of the centromeres and telomeres in *B. distachyon* leaf nucleus presented in Fig. 2F.

Supplementary Video S13. The non-Rabl organization of the centromeres and telomeres in *B. stacei* in the small spherical nucleus of the root tip that is presented in Fig. 4A.

Supplementary Video S14. The non-Rabl organization of the centromeres and telomeres in *B. stacei* in the small elongated nucleus of the root tip that is presented in Fig.4B.

Supplementary Video S15. The non-Rabl organization of the centromeres and telomeres in *B. stacei* in the rod-shaped nucleus of the root tip that is presented in Fig. 4C.

Supplementary Video S16. The non-Rabl organization of the centromeres and telomeres in *B. hybridum* in the elongated endoreduplicated nucleus of the root tip that is presented in Fig. 4E.

Supplementary Video S17. The Rabl organization of the centromeres and telomeres in *B. hybridum* in the spherical endoreduplicated nucleus of the root tip that is presented in Fig. 4D.

Supplementary Video S18. The Rabl organization of the centromeres and telomeres in *B. stacei* in the elongated endoreduplicated nucleus of the root tip that is presented in Fig. 4F.

Supplementary Data
