# Supplementary material for: Spatial distribution of centromeres and telomeres at interphase varies among Brachypodium species
Source: J Exp Bot. 2015 Jul 24;66(21):6623–34. doi: 10.1093/jxb/erv369 (PMC4623680; doi:10.1093/jxb/erv369)
Supplement: Supplementary Data [file supp_66_21_6623__index.html]

Spatial distribution of centromeres and telomeres at interphase varies among Brachypodium species — Spatial distribution of centromeres and telomeres at interphase varies among Brachypodium species — Supplementary Data 

# Spatial distribution of centromeres and telomeres at interphase varies among *Brachypodium* species

## Supplementary Data

Data files

- Supplementary Data - Supplementary Data
- Supplementary Data - Supplementary Data
- Supplementary Data - Supplementary Data
- Supplementary Data - Supplementary Data
- Supplementary Data - Supplementary Data
- Supplementary Data - Supplementary Data
- Supplementary Data - Supplementary Data
- Supplementary Data - Supplementary Data
- Supplementary Data - Supplementary Data
- Supplementary Data - Supplementary Data
- Supplementary Data - Supplementary Data
- Supplementary Data - Supplementary Data
- Supplementary Data - Supplementary Data
- Supplementary Data - Supplementary Data
- Supplementary Data - Supplementary Data
- Supplementary Data - Supplementary Data
- Supplementary Data - Supplementary Data
- Supplementary Data - Supplementary Data
- Supplementary Data - Supplementary Data
